# Fast detection of unique genomic regions

**DOI:** 10.1016/j.csbj.2025.02.025

**Published:** 2025-02-27

**Authors:** Beatriz Vieira Mourato, Bernhard Haubold

**Affiliations:** Research Group Bioinformatics, Max-Planck-Institute for Evolutionary Biology, August-Thienemann-Str. 2, 24306, Plön, Germany

## Abstract

Unique genomic regions are of particular interest in two scenarios: When extracted from a single mammalian target genome, they are highly enriched for developmental genes. When extracted from target genomes compared to closely related neighbor genomes, they are highly enriched for diagnostic markers. Despite their biological importance and potential economic value, unique regions remain difficult to detect from whole genome sequences. In this review we survey three efficient programs for the detection of unique regions at scale, genmap, macle, and fur. We explain these programs and demonstrate their application by analyzing simulated and real data. Example scripts for searching for unique regions are available from the Github repository evolbioinf/sure as part of a detailed tutorial.

## 1 Introduction

Mammalian genomes are famously repetitive, and mammalian genome browsers routinely display their dense tracks of repeats. These repeats are found by homology to known repeats. Non-repetitive, or unique, regions are less well understood, as they often cannot be directly annotated by homology. Still, unique regions have attracted considerable interest, mostly in the form of genetic markers. Genetic markers are regions common to a set of target genomes that are absent from all other genomes—their uniqueness is inter-genomic. The markers amplified in PCR tests for Covid-19 are perhaps the best-known examples.

Markers are a special case of inter-genomic uniqueness. If they occur across a monophyletic target clade, they are part of the set of lineage-specific regions of that clade. In practice, it is often hard to distinguish genetic variation between individuals from variation between lineages. Consider two lineages, *A* and *B*, each represented by a single genome, $a_{1}$ and $b_{1}$. Any region, *r*, in $a_{1}$ absent from $b_{1}$ might appear specific for *A*. However, a second genome sampled from *A*, $a_{2}$, might well not contain *r*, revealing it to be part of the genetic variation between individuals in *A* rather than between lineages *A* and *B*. In other words, substantial sampling is often key for reliably identifying lineage-specific regions. Still, even in the days when only a single human and chimp genome were available, human- and chimp-specific regions were a major focus of their analysis [1]. This only goes to show the intense interest in lineage-specific regions in the great apes and other mammals.

Apart from the mainstream interest in markers and lineage-specific regions, there is also some work on uniqueness within individual genomes, intra-genomic uniqueness. This is due to the observation that regions unique within individual mammalian genomes are highly enriched for developmental genes [2], [3], [4].

Regardless of whether the regions of interest are unique within or between genomes, the tools for finding them are less well established than for finding similar regions. The need for specialized tools for finding unique regions may appear surprising in the first place, as conceptually it is easy to identify unique regions: Carry out a similarity search and take its complement. As we shall see, detecting unique regions on the scale of genomes is more involved, but similarity searches are a good starting point for our exploration of methods for finding unique regions.

Bioinformatics is founded on sequence similarity searches. Gusfield's “first fact of sequence analysis”, which posits that similar sequences have similar function, explains this preeminence of sequence similarity searches in bioinformatics: they facilitate, if not replace, experiments when annotating sequences [5, p. 212]. Apart from sequence annotation, sequence similarity searches underlie most work in comparative genomics, ranging from *de novo* genome assembly based on overlapping shotgun reads to phylogeny reconstruction based on multiple sequence alignments.

Among the most successful tools for sequence similarity search are the programs of the Blast package [6], [7], [8]. They are intended for comparing one or more short query sequences to a large collection of potentially highly diverged subject sequences. However, this asymmetrical search across large evolutionary distances does not fit the context in which searches for unique regions are most fruitful. The search for lineage-specific regions, and hence also for markers, works best when comparing a sample of target genomes to a sample of closely related, but distinct, neighbor genomes [9].

**Fig. 1:**
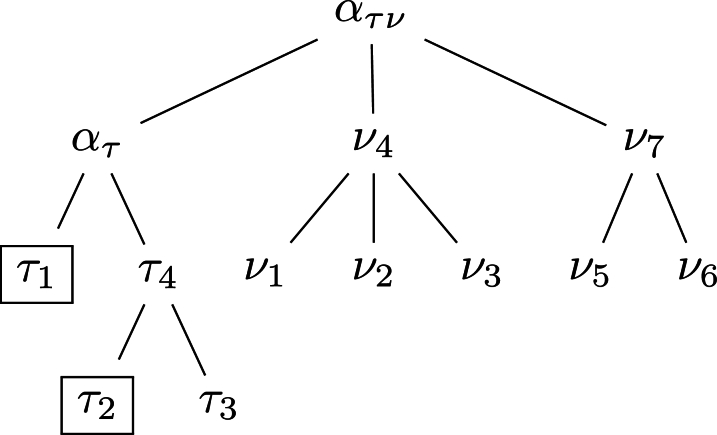
Detecting targets and neighbors in a taxonomy. Starting from the boxed target taxa *τ*_1_ and *τ*_2_, we visit their most recent common ancestor, *α*_*τ*_, which implies an additional two targets, *τ*_3_ and *τ*_4_. By visiting the parent of *α*_*τ*_, *α*_*τν*_, the neighbors *ν*_1_,...,*ν*_7_ are discovered as the taxa in the tree rooted on *α*_*τν*_ minus the taxa in the tree rooted on *α*_*τ*_, minus *α*_*τν*_.

This begs the question, what are the neighbors of a set of target taxa, T? One way to find them is based on the most recent common ancestor of T, as illustrated in Fig. 1 [10]. Let $\tau_{1}$ and $\tau_{2}$ be the original targets. Their most recent common ancestor, $\alpha_{\tau }$, implies two more targets, $\tau_{3}$ and $\tau_{4}$, yielding the target set $T=\{\tau_{1},...,\tau_{4},\alpha_{\tau }\}$. The parent of $\alpha_{\tau }$, $\alpha_{\tau \nu }$, defines the set of taxa $A=\{T,\nu_{1},...,\nu_{7},\alpha_{\tau \nu }\}$. We extract the neighbors from A by subtracting the targets and their parent,

$$N=A\setminus T\setminus \alpha_{\tau \nu }.$$

However, it is well known that taxonomy does not always agree with the underlying phylogeny. Hence the taxonomic targets and neighbors just found still need to be sorted into their phylogenetic equivalents by fast phylogeny reconstruction. Regions that are ubiquitous in the genomes of the resulting phylogenetic targets but absent from the genomes of the phylogenetic neighbors are candidate lineage-specific regions and/or diagnostic markers.

This approach to discovering diagnostic markers is rooted in evolutionary thinking. Evolution implies that the vast majority of regions that are similar to the targets—and hence *not* unique—is contained in the targets' closest relatives. Removing all target material with homology to the neighbors strongly enriches for universally unique, and hence diagnostic, regions.

As we already alluded to in the context of lineage-specific regions, the uniqueness of a target region with respect to neighbors is contingent on the target and neighbor genomes available. A new target genome might not contain a given unique region, thereby violating the ubiquity criterion. Similarly, a new neighbor might contain a hitherto unique region, thereby violating the absence criterion. As a consequence, inter-genomic uniqueness needs to be rechecked from time to time. To facilitate this, the task of finding unique regions ought to encompass a convenient method for finding target and neighbor genomes. This is accomplished by using the tree-climbing strategy outlined in Fig. 1, combined with fast phylogeny reconstruction. This is implemented in the programs of the Neighbors package [10].

**Fig. 2:**
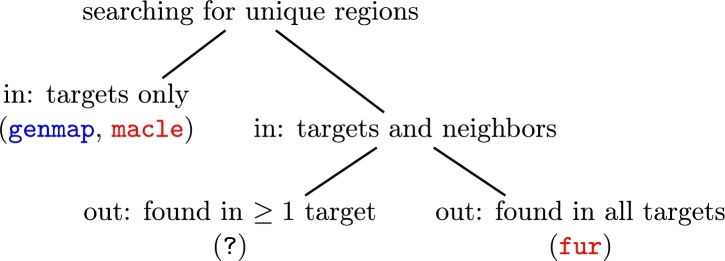
How to search for unique regions. Unique regions can be extracted from input (*in*) that consists either only of a sample of target sequences or of two samples, targets and neighbors. When working with targets and neighbors, the unique regions output (*out*) may either be found in at least one target or in all targets. Programs shown in red are based on maximal matches, the program in blue on k-mers; the *?* indicates a “missing” program.

This review is a practical guide to finding suitable samples of genome sequences and detecting unique regions from them. To structure the review, we have already distinguished intra- and inter-genomic uniqueness. In Fig. 2 we make this nomenclature more succinct by distinguishing between two types of input, targets only, for finding intra-genomic uniqueness, or targets and neighbors, for finding inter-genomic uniqueness.

Assuming we can find suitable genomes using a package like Neighbors, what we still need, are tools for efficiently extracting unique regions from them. These are surprisingly hard to come by. For example, the program Kec, for “k-mer exclusion”, has been used successfully to identify unique regions from bacterial genomes in the same target/neighbor constellation we are investigating here [11]. However, when testing Kec, we found that a significant part of its “unique” output can still be found among the neighbors. This means *in vitro* testing of the marker candidates returned by Kec is essential to guard against false positives. Similarly, a recent phylogeny-guided method for finding unique regions in microbial genomes returns target regions with 92% similarity in neighbors, again making their specificity dependent on *in vitro* testing [12]. We settled on the three tools we could find that combined efficiency with high *in silico* specificity, genmap [13], macle [3], and fur [14], [10].

As explained in more detail in the Methods section, the programs we survey are based on two kinds of matches, fixed length matches called k-mers, and variable length matches bounded by mismatches called maximal matches. The program genmap is based on k-mers and shown in blue in Fig. 2, the programs macle and fur are based on maximal matches and shown in red.

The program genmap is widely used to calculate a generalized version of mappability, the “(k,e)-mappability”. This is defined for each position of a genome as the inverse of the number of k-mers starting at that position found across the sequence with no more than *e* mismatches [13]. So a unique k-mer induces a mappability of 1, a highly repeated k-mer a mappability close to zero. Regions with high mappability are unique.

Mappability with mismatches is an important quantity in genomics. During read mapping, genome regions with low mappability attract reads from more than one genomic region. This results in unreliable estimates of local polymorphism levels. As a consequence, many population genomic studies are restricted to regions of high mappability. For studies based on 150 bp reads, for example, it is customary to only analyze regions with high (150,2) mappability [15], [16].

Unfortunately, the mappability threshold for uniqueness is not known, but it should be possible to discover it using Monte Carlo simulation. Given such a mappability threshold, we can pick unique windows from a sliding window analysis. Overlapping unique windows are then merged into the desired unique regions. In humans and mice unique regions were first shown almost twenty years ago to be highly enriched for developmental genes [2].

One advantage of the program macle is that it comes with a method for calculating a uniqueness threshold as a function of sequence length and GC content. Based on maximal matches, macle calculates the “match complexity”, $C_{m}$, which is defined for sliding windows of, say, 10 kb. The $C_{m}$ ranges from zero for a window repeated exactly elsewhere, to an expectation of 1 for a window where the match lengths cannot be distinguished from the random null distribution.

**Fig. 3:**
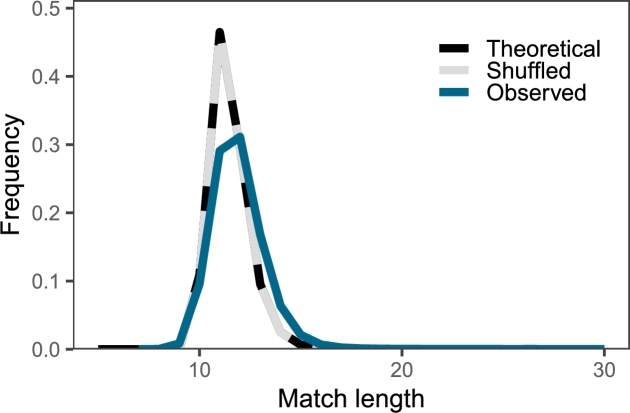
The observed distribution of match lengths in the genome of *E. coli* K12 (*Observed*) compared to its theoretical null distribution (*Theoretical*) and the null distribution simulated by shuffling the genome (*Shuffled*); note that the shuffling agrees so closely with the theory that their graphs are superimposed; please note also that the match lengths observed in *E. coli* K12 range from 7 to 3027—we only plotted range 7–30 to keep the graph intelligible.

The null distribution of $C_{m}$ [3] is based on the null distribution of match lengths, which has been known for over 15 years [17]. Fig. 3 shows the distribution of match lengths observed in the genome of *E. coli* K12 compared to its theoretical null distribution and the null distribution simulated by shuffling the genome. As expected from the underlying theory [17], the theoretical and simulated curves are indistinguishable.

Given the null distribution of $C_{m}$, we can calculate a $C_{m}$ threshold and use it like the mappability threshold to pick unique regions. In a recent study, unique regions detected with $C_{m}$ in 18 mammalian genomes sampled from placentals and marsupials were shown to be highly enriched for developmental genes [4], thus agreeing with earlier work in humans and mice [2], [3].

**Fig. 4:**
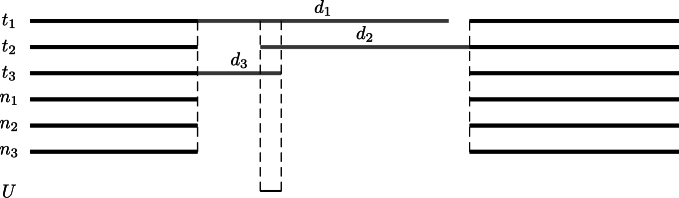
Cartoon of a multiple sequence alignment of three target sequences, *T* = {*t*_1_,*t*_2_,*t*_3_} and three neighbor sequences, *N* = {*n*_1_,*n*_2_,*n*_3_}; their difference, *D* = *T* − *N*, consists of the three gray regions, *D* = {*d*_1_,*d*_2_,*d*_3_}, and the regions unique to *T* consist of their intersection *U* = {*d*_1_ ∩ *d*_2_ ∩ *d*_3_}.

If we move on from unique regions in sets of targets to comparisons between target and neighbor sequences, Fig. 2 further subdivides their analysis by output: The unique regions uncovered from targets and neighbors might be present in at least one target or in all targets. As to presence in at least one target, consider, for example, the alignment in Fig. 4 consisting of three target sequences $T=\{t_{1},t_{2},t_{3}\}$ and three neighbor sequences $N=\{n_{1},n_{2},n_{3}\}$. The difference between targets and neighbors, D=T−N, consists of the three gray regions $D=\{d_{1},d_{2},d_{3}\}$. This difference could be calculated by generalizing the application of the target-only programs genmap or macle, but we know of no dedicated program to reliably carry out this task, hence the question mark in Fig. 2.

The regions present in all targets are obtained by intersecting the elements of *D* to get the unique target regions, in our example the single region $U=\{d_{1}\cap d_{2}\cap d_{3}\}$. This is what fur does [10].

In the following we explain the salient features of the three programs we survey, genmap, macle and fur. We then apply them to simulated and real data. For each of the three programs we show how simulation can be used to construct data sets with known unique regions, which serve as positive controls.

As an example of a real target for finding intra-genomic unique regions, we take the shortest human chromosome, chromosome 21, which is 46.7 Mb long. To find an example of a comparison between targets and neighbors, we looked through a list of 120 reference bacteria that were recently surveyed for markers [10]. We singled out taxa with a moderate number of genomes to allow easy replication. Apart from that, the ideal demo taxon would also display disagreement between taxonomy and phylogeny, a common problem in the analysis of bacterial genomes already mentioned.

We settled on the bacterial pathogen *Legionella pneumophila*, the cause of Legionnaires' disease [18]. *L. pneumophila* consists of three subspecies, *pneumophila*, which contains the type strain Philadelphia 1, *fraseri*, and *pascullei* [19]. We concentrated on subspecies *pneumophila*, which at the time of writing comprised 42 target and neighbor genomes, whose phylogeny did not always follow their taxonomy; in the Results section we explain how to handle such discrepancies between taxonomy and phylogeny.

We emphasize practical application, and our Github repository Sure, for “searching for unique regions”, contains a tutorial on the central analyses used in this review together with scripts for replication and experimentation. It is located at github.com/evolbioinf/sure

## 2 Methods

Table 1 summarizes the approaches of the three programs we explore, the number of target and neighbor sequences they take as input, and the output they generate. Notice that only fur actually outputs unique regions, while the output of genmap and macle—mappability and complexity ($C_{m}$), respectively—still needs to be converted into such regions. We explore the three programs along their division by input into targets only and targets/neighbors shown in Fig. 2.

**Table 1:** The three programs for finding unique genome regions surveyed.

Program	Matches	Input		Output
		Query	Subject	
genmap[13]	k-mer	≥1	0	mappability
macle[3]	maximal	≥1	0	match complexity, *C*_m_
fur[10]	maximal	≥1	≥1	unique regions

### 2.1 Targets only

The programs genmap and macle take an arbitrary number of sequences as input, but make no distinction between them, in effect treating them like one long target.

*genmap.*  The program genmap takes as input a sequence and counts the k-mers with up to *e* mismatches starting at every position in it. For example, given the target t=TAAATAAAATTT, k=3, and e=0, Table 2 shows the k-mer counts for the forward strand. The first 3-mer, TAA, appears twice in *t*, starting at positions 1 and 5; the second 3-mer, AAA, appears three times at positions 2, 6, and 7.

**Table 2:** K-mer counts and mappability with *k* = 3 and no mismatches along *t* = TAAATAAAATTT.

position	1	2	3	4	5	6	7	8	9	10	11	12
sequence	T	A	A	A	T	A	A	A	A	T	T	T
count	2	3	2	1	2	3	3	2	1	1	–	–
mappability	0.5	0.3	0.5	1.0	0.5	0.3	0.3	0.5	1	1	–	–

To efficiently count the (k,e)-matches, the authors of genmap combined three algorithmic innovations: (i) based on bidirectional FM indices, they applied optimum search schemes to find all approximate (k,e)-matches; (ii) they summarized overlapping k-mers by their common substrings and searched for that common core before extending it to the left and to the right; (iii) they searched for each distinct k-mer only once [13]. Together, these three algorithmic modules make genmap orders of magnitude faster than its predecessor, GEM-tools [20]. The actual output of genmap is the inverse of these counts, the sequence's (k,e)-mappability.

*macle.*  Instead of counting k-mers, the program macle calculates the lengths of all right-maximal matches in a sequence, *S*. These lengths are looked up from the sorted set of suffixes in *S*, the suffix array of *S*. This suffix array is enhanced by the lengths of common prefixes and by child nodes to yield an abstraction of a suffix tree of *S* [21, ch. 4].

**Fig. 5:**
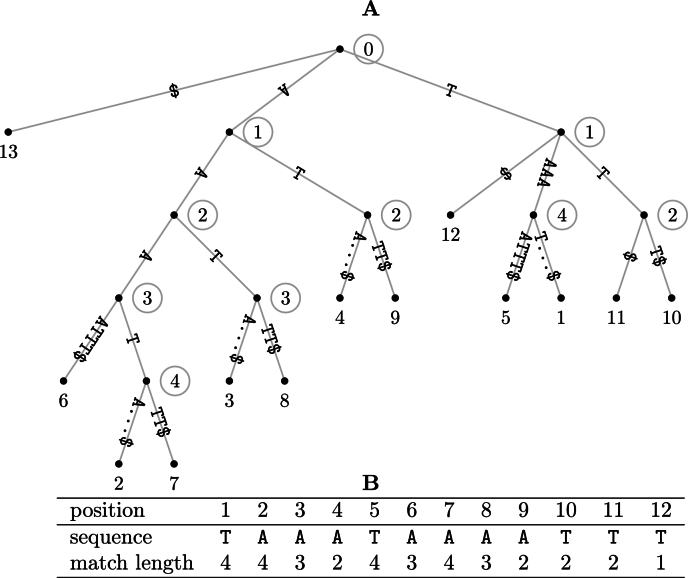
Suffix tree (**A**) of the target DNA sequence *t* = TAAATAAAATTT consisting of twelve nucleotides and a terminal sentinel character, $; a leaf label *i* refers to the suffix *t*[*i*...], a circled node label denotes the length of the path label from the node, the node's “string depth”. The suffix tree allows the calculation of match lengths (**B**) in *t* by noting the string depth of each leaf's parent.

Fig. 5A shows the suffix tree for our example target sequence, *t*. There is a leaf for each of the |t| suffixes, and each path from the root to a leaf spells out the suffix starting at the leaf label. For example, leaf 6 has the path label

t[6...]=AAAATTT$,

where the terminal $ mismatches any character in the input sequence [5, p. 90f].

In the suffix tree, matching prefixes of the suffixes are summarized along paths leading to internal nodes. For example, the parent node of leaf 6 has path label AAA, which is the common prefix of suffixes t[6...], t[2...], and t[7...]. The circled 3 next to that node indicates the length of its path label, its “string depth”. Since the root is the only node without a parent, it is also the only node with string depth zero. We observe that internal nodes are labeled with matches, while labels ending on terminal branches are unique. This suggests a simple method for looking up all right-maximal match lengths in a sequence: traverse its suffix tree and at every leaf, *i*, note the string depth of its parent as the match length ml[i]. Fig. 5B shows the match lengths for *t* looked up like this.

### 2.2 Targets and neighbors

The program fur carries out three steps to find the target regions that are unique with respect to a sample of neighbor genomes:

1. Subtraction 1: Subtract the neighbors from an arbitrary reference target
2. Intersection: Intersect all other targets with the output from Subtraction 1
3. Subtraction 2: Use Blast to remove residual neighbor material from the output of Intersection

Subtraction 1 and Intersection both rely on maximal matches to determine homology. In Subtraction 1, fur calculates the lengths of the longest matches between the reference target and the neighbors starting at every position in the reference. Like in the analysis of the single sequence, these match lengths are calculated based on enhanced suffix arrays, and reference regions with short matches are picked as unique. In the Intersection, these reference regions are intersected with all other targets to determine the common regions. In Subtraction 2, finally, any neighbor material remaining in the common regions is removed with Blast in “blastn” mode and *E*-value $10^{-5}$.

To recap the methods overview, let's return to Table 1, where our three programs are listed. The binary division into k-mer and maximal match algorithms is readily apparent. Similarly, we see that their output is divided into two categories, local statistics from which unique regions still have to be extracted, and actual unique regions. To further clarify the difference, we apply the programs to simulated and real data.

### 2.3 Simulated data

The program stan from the Github repository evolbioinf/stan simulates targets and neighbors, hence its name. The targets contain one or more regions missing from all the neighbors, the marker regions.

**Fig. 6:**
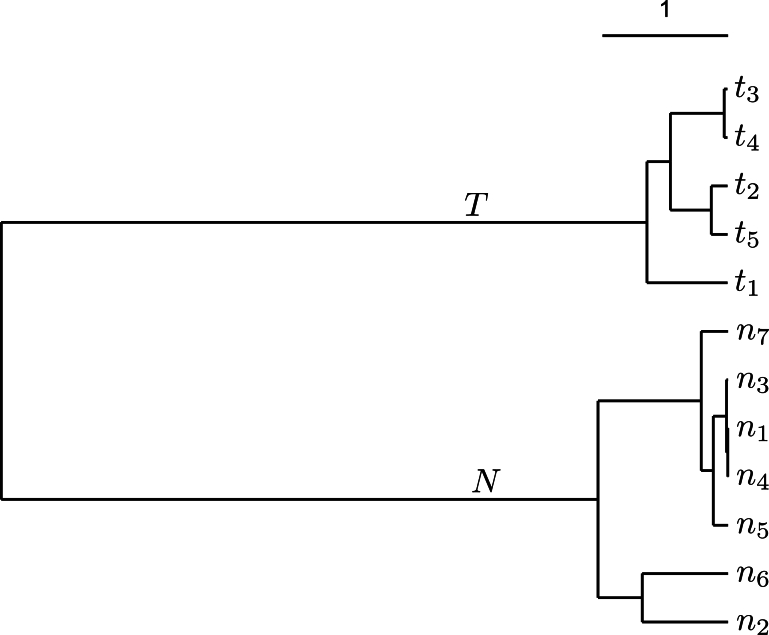
Example coalescent for 5 targets, *T* = *t*_1_,*t*_2_,...,*t*_5_, and 7 neighbors, *N* = *n*_1_,*n*_2_,...,*n*_7_.

Under the hood, stan simulates the genealogies of the targets and neighbors separately, before the two genealogies coalesce back in time. In evolutionary biology, such time-reversed genealogies are known as coalescents [22], and Fig. 6 shows an example with 7 targets and 13 neighbors.

### 2.4 Real data

#### 2.4.1 Human chromosome 21

We downloaded the assembly of the human reference genome, accession GCF_000001405.40, and the corresponding annotation file from the Entrez database run by the NCBI using their datasets tool. Chromosome 21 has accession NC_000021.9 in that assembly.

#### 2.4.2 *Legionella pneumophila* genomes

We identified the currently available 42 target and neighbor genomes for *Legionella pneumophila* using the neighbors program from the Github repository evolbioinf/neighbors starting with target *L. pneumophila* subsp. *pneumophila* (taxon-ID 91891). The 42 genomes are listed in Table S1 and were downloaded again using datasets.

### 2.5 Resource consumption

With one exception, time and memory consumption was measured under the Windows Subsystem for Linux with Windows 11 using the program /usr/bin/time on a laptop equipped with 16 12th Gen Intel i7 processors and 32 GB RAM. The exception was the time-consuming survey of (k,e)-mappabilities summarized in Table 3. These runs were carried out with 64 threads on our server running 64 Intel Xeon processors with 256 GB RAM under Ubuntu 24.04. The run times on this machine are “user” times to account for multi-threading.

**Table 3:** Yield of unique regions, run times (*t*), and specificity (*s*_p_) of various (*k*,*e*)-mappabilities calculated with genmap for human chromosome 21.

*k*	*e*	yield (kb)	*s*_p_	*t* (s)
15	0	0	—	60
15	1	2,048	0.89	947
15	2	4,793	0.65	13,614
30	0	918	0.99	48
30	1	277	1.00	241
30	2	86	1.00	934
50	0	7,256	0.90	42
50	1	3,573	0.95	134
50	2	2,007	0.98	241
100	0	31,847	0.77	44
100	1	29,297	0.79	117
100	2	25,452	0.80	137
150	0	33,596	0.75	45
150	1	32,719	0.77	125
150	2	32,055	0.77	143

## 3 Applications

### 3.1 Resource consumption

The three programs we survey, genmap, macle, and fur, are designed for efficiency. They also operate on indexes, so we measured their time and memory consumption separately for index construction and index analysis, or “mapping”. As input we simulated one target and ten neighbor sequences of equal length.

**Fig. 7:**
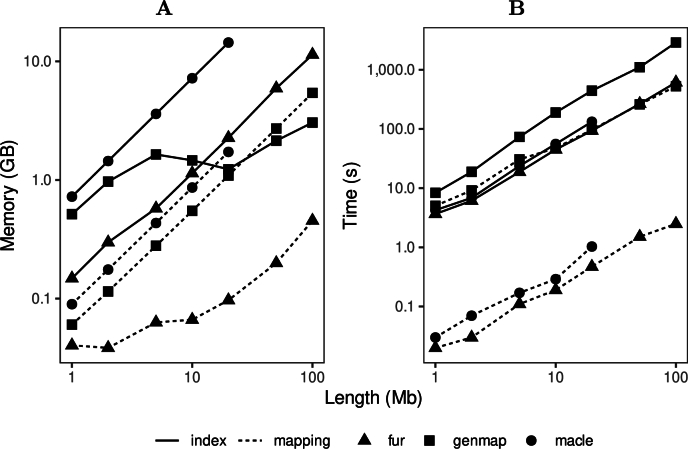
Single-threaded memory (**A**) and time (**B**) consumption of the three programs we survey, divided into their indexing (solid line) and mapping (dashed line) steps.

Fig. 7A shows the single-threaded memory consumption as a function of sequence length ranging from 1 Mb to 100 Mb. The indexing programs are shown with solid lines, the mapping programs with dashed lines. Most memory consumption is approximately linear in the input size. The exception is the indexing step of genmap. Its memory consumption is flat between sequence lengths 5 Mb and 20 Mb, only to pick up again beyond that. The program macle consumes so much RAM that it runs out of memory on our testing laptop for samples with sequences of lengths 50 Mb and beyond. This illustrates the importance of memory-efficient indexing as implemented in genmap [13] and fur [10].

Fig. 7B shows the run times when applied to the same input. Again, the macle graphs don't extend beyond samples of 20 Mb sequences, as the program ran out of memory beyond. Like memory consumption, run time is approximately linear in input size with mapping faster than indexing, five times faster for genmap, two orders of magnitude faster for macle and fur.

### 3.2 Targets only

**Fig. 8:**
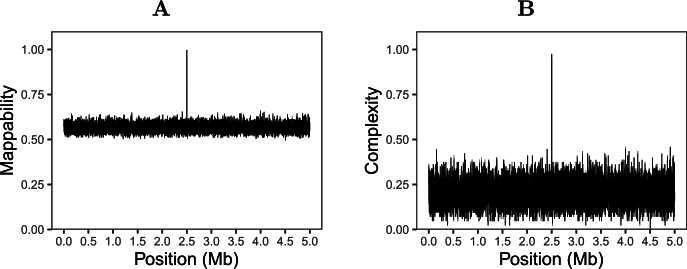
The (15,0)-mappability with k-mers of length 15 (**A**) and complexity (**B**) in 1 kb sliding windows along a random 5 Mb sequence on the background of a homologous sequence diverged by 1 %; the background sequence also has a 2 kb deletion at its center, which shows up as the peak of unique sequence in the middle.

We start our analysis of targets by simulating a 5 Mb duplication that has diverged by 1%. This mimics two *E. coli* genomes. In the middle of the duplication we insert 2 kb of random sequence, the desired unique region. Fig. 8A shows the (15,0)-mappability in 1 kb windows along the simulated sequence. The mappability hovers around 0.57. To account for this, we note that all k-mers in our simulation ought to appear twice, once in the original sequence and once in the duplication, unless they have mutated, in which case they only appear once. The probability of a k-mer mutating is the complement of it not mutating,

$$P_{m}=1-P_{0},$$

where $P_{0}={\left(1-\mu \right)}^{k}$ and *μ* is the mutation rate. So we can estimate the expected mappability as $P_{0}/2+P_{m}$, which for our μ=0.01 and k=15 is ≈0.57, close to the result of the simulation.

The spike in the middle of the graph indicates the 2 kb unique region. Given the excellent separation of signal and noise in our simulation, it is easy to pick the unique region using a sliding window analysis.

Fig. 8B shows the complexity, $C_{m}$, along the same data. The $C_{m}$ hovers around 0.21 with a larger variance than the mappability. However, the spike pointing to the 2 kb unique region is again well separated from the background.

With a length of 46.7 Mb, chromosome 21 is the shortest of the human chromosomes, which is why we chose it as our example for analyzing a real target sequence. Computation of its genmap index took 56 s and 1.4 GB of RAM. Calculating the (15,0)-mappability then took another 20 s and 0.3 GB of RAM, followed by a 10 kb sliding window analysis. To distinguish unique from non-unique regions, we looked up the 5% mappability quantile from a shuffled version of chromosome 21. However, no window in the real data reached this threshold. So we tried other combinations of *k* and *e*. As summarized in Table 3, we calculated mappabilities for k={15,30,50,100,150}. For each value of *k* we used e={0,1,2}. Most runs took of the order of 10^2^ s, the biggest exception being the (15,2)-mappability, which threw up so many matches that it took 13,614 s (Table 3).

The yields of these runs varied between nothing and 33.6 Mb (Table 3). This prompted us to calculate the specificity of the uniqueness prediction as the fraction of nucleotides not found by Blast outside the region of origin (true positive). The specificity varied from 0.65 for (15,2)-mappability to 1 for (30,1)- and (30,2)-mappability (Table 3). We decided to accept a specificity of at least 0.95, and to then pick the combination of *k* and *e* with the highest yield. This was the (50,1)-mappability with specificity 0.95 and yield totaling 3.6 Mb.

Indexing chromosome 21 with macle took 18 s, a third of the run time of genmap, and 3.1 GB RAM, twice the amount used by genmap. The sliding window analysis of the macle index then took a mere second, compared to 134 s for (50,1)-mappability with genmap, and 0.4 GB RAM, a third more than genmap.

Using the $C_{m}$ threshold for unique regions, 0.9951, we found 33 unique regions covering a total of 442 kb, just under 1% of chromosome 21. The specificity of the macle prediction was 0.99, well above the 0.95 specificity threshold we deemed acceptable.

Given that we are using mappability and complexity to predict the same thing, uniqueness, the 442 kb predicted from complexity ought to be contained in the 3.6 Mb predicted from (50,1)-mappability. In fact, only 81% of the complexity regions were contained in the mappability regions. There were four complexity regions with zero overlap summing to 43 kb with a specificity of 0.97. So mappability and complexity return somewhat different unique portions of a given sequence.

**Fig. 9:**
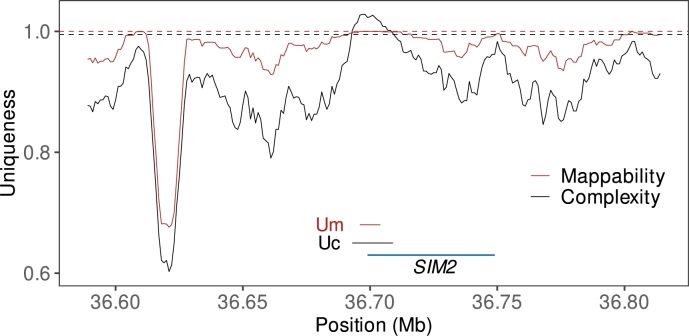
Two measures of uniqueness along a portion of human chromosome 21, *Complexity* and (50,1)-*Mappability*; the dashed horizontal lines are the thresholds, which happen to be almost identical; shown are also the positions of the *SIM2* gene and of the similar unique regions identified on the basis of the complexity track, *Uc*, and on the basis of the mappability track, *Um*.

To make the most of the two methods, we looked for the two regions with the longest intersection between them. This was 18 kb long and located at position 36.7 Mb. Fig. 9 shows the mappability and complexity along 200 kb around this region. The two unique regions predicted intersect one protein-coding gene, *SIM2*.

“*SIM2*” stands for “single-minded 2”, and the gene was originally named for the morphological effect of its knockout on the mid-line neuroepithelium of fruit flies, where two strands of neurological tissue merge into one [23]. *SIM2* encodes a transcription factor based on the helix-loop-helix motif and mouse knock-outs are associated with severe morphological and neurological abnormalities [24]. In humans, the gene is located in the region on chromosome 21 critical for the development of Down syndrome [25]. And while the exact molecular details of *SIM2* action during development and beyond are still not entirely clear, it is known that Down syndrome patients have numerous amino acid changes in the SIM2 protein that *in vitro* impair its function as a transcription factor [24].

### 3.3 Targets and neighbors

**Fig. 10:**
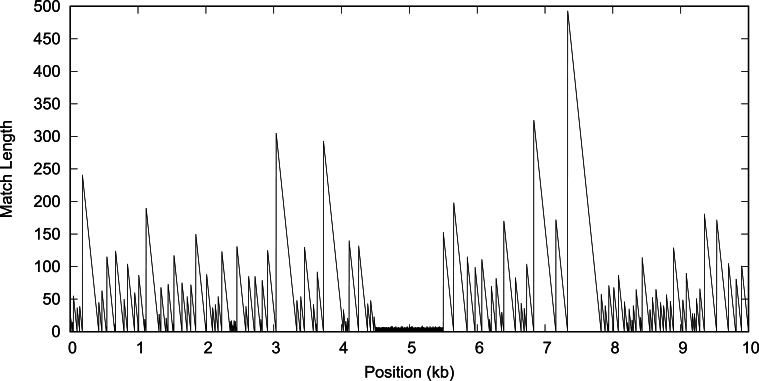
The match length along a simulated 10 kb pair of sequences, where the target contains a centered 1 kb insertion marked by the short matches.

We now expand our analysis of targets to a comparison between targets and neighbors to find unique target regions. Again, we begin with simulations, this time by simulating 10 kb target and neighbor sequences along their genealogy, or coalescent (Fig. 6), and inserting a 1 kb unique region into all targets with the program stan. Fig. 10 shows the match length along the target. We see the jagged regions of long, homologous matches that bracket the central 1 kb containing short, random matches. Like macle before, fur uses this contrast between regions with long and short matches to pick unique regions.

We next investigated the accuracy of fur from simulated data. The null distribution of match lengths underlying fur (Fig. 3) is strictly speaking only correct in the limit of infinite sequence length [17]. Hence we investigated accuracy as a function of input size, specifically sequence length and neighborhood size. As accuracy measure we used the correlation between prediction and ground truth developed to assess gene prediction programs, *C* [26, p. 121f]. Like any correlation, *C* ranges from -1 to 1, with 1 indicating perfect prediction and -1 indicating a prediction where each nucleotide classified as part of a unique region is in fact part of a repeat, and vice versa. Fig. S1A shows *C* as a function of sequence length ranging from 10 kb to 10 Mb when comparing one target and one neighbor. For 10 kb *C* is 0.71 but then rises to values oscillating around 0.98. When varying the number of 10 kb neighbors in Fig. S1B, *C* is below 0.75 for 1 and 2 neighbors, but jumps to 0.99 for 5 neighbors and eventually reaches 1. In other words, beyond very small inputs, fur reliably picks unique regions.

**Fig. 11:**
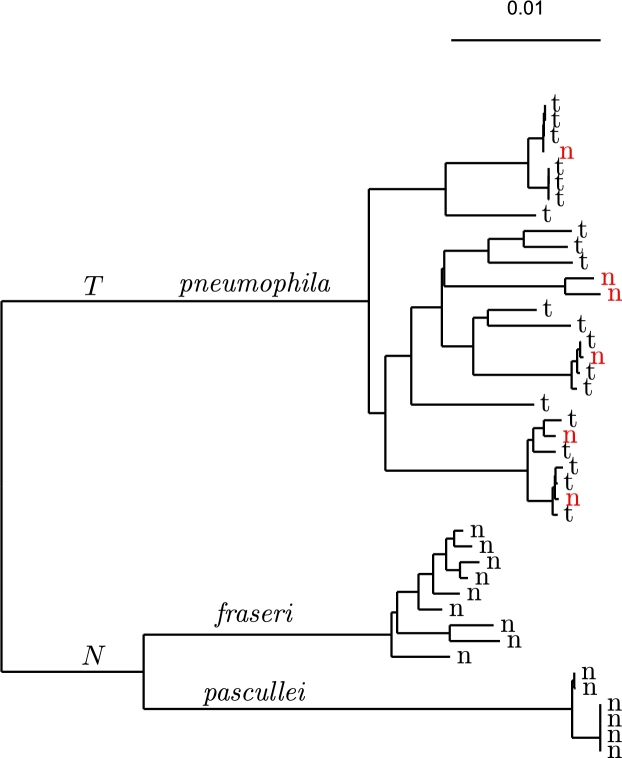
Phylogeny of taxonomic targets (t) and neighbors (n) for target *Legionella pneumophila* subsp. *pneumophila*; the phylogenetic targets (*T*) contain six taxonomic neighbors in red that don't cluster with the phylogenetic neighbors (*N*) consisting of the two subspecies *fraseri* and *pascullei*.

As an example for real target/neighbor data, we investigated the bacterium *Legionella pneumophila*. This human pathogen is transmitted via the water system and as a result is subject to intense monitoring efforts, including by diagnostic PCR [18]. At the time of our analysis, there were 21 complete target and also 21 complete neighbor genomes available (Table S1). Fig. 11 shows the phylogeny of the 42 genomes. Its three clades correspond to the three subspecies of *L. pneumophila*, our target *pneumophila*, and the neighbors *fraseri*, and *pascullei* [19]. Each leaf of the phylogeny is just labeled “t” or “n” to denote target or neighbor. Notice the six neighbor genomes in red among the subsp. *pneumophila* targets, *T*. We take them to be misclassified subsp. *pneumophila* and hence end up with 27 targets and 15 neighbors.

The genome sequences of targets and neighbors were converted to a fur database using the program makeFurDb, which took 18 s and 488 MB of RAM on a single thread. When using all available 16 threads on our testing laptop, the run time dropped by 50% to 13 s and the memory requirement grew sixfold to 3.3 GB.

The analysis of this database with the program fur took another 6 s and 257 MB RAM to yield 24 fragments ranging in size between 108 bp—close to the default minimum of 100—and 1,273 bp, totaling 9,117 bp.

It is important to realize that membership in these 9,117 bp is not sufficient to be diagnostic, horizontal gene transfer might have transferred target material beyond the neighbors. However, it is necessary. In other words, any marker that can be discovered, is part of these unique 9,117 bp uncovered in a target/neighbor comparison.

As already noted in the Introduction, this analysis of *L. pneumophila* was part of a sample of 120 bacterial strains that were recently subjected to large-scale marker discovery [10]. In that analysis, all marker candidates were also subjected to automatic primer construction. The primers were then tested for sensitivity and specificity through *in silico* PCR on the non-redundant collection of nucleotide sequences distributed by the NCBI. In the case of *L. pneumophila* subsp. *pneumophila*, the result was a pair of primers with perfect *in silico* sensitivity and specificity. Such a pair of primers could next be tested *in vitro* before deployment in real-world diagnostics.

## 4 Conclusions

Unique genomic regions are enriched for developmental genes and diagnostic markers. Traditionally, the problem of searching for unique genomic regions has been solved using tools for similarity search. Although this does not scale well when applied to whole genome sequences, few dedicated programs exist for finding unique regions. In this review we surveyed three such programs, genmap, macle, and fur, picked for their efficiency and *in silico* accuracy. For genmap we showed that the yield of unique regions is highly dependent on the choice of parameters *k* and *e*. It would be helpful to develop theory for finding the optimal combination of *k* and *e* for detecting uniqueness.

Such theory exists for the lengths of maximal matches underlying macle. As a result, it has excellent specificity, though it finds less material than deemed unique from Blast runs. However, we ran Blast in default mode, which is based on the low-sensitivity megablast algorithm, so homology might have been under-reported. Of the three programs surveyed, fur is the only one that directly returns unique regions. Its accuracy is excellent, unless the data set analyzed is too small (Fig. S1). Together with the accompanying tutorial, this clears the way for convenient detection of unique regions both from single target genomes and from pairs of samples of target and neighbor genomes.

## Funding

We received generous core funding from the 10.13039/501100004189Max Planck Society.

## CRediT authorship contribution statement

**Beatriz Vieira Mourato:** Writing – review & editing, Visualization, Software, Methodology, Data curation, Conceptualization. **Bernhard Haubold:** Writing – original draft, Software, Methodology, Data curation, Conceptualization.

## Declaration of Competing Interest

None.

## Appendix A Supplementary material

MMC This Supplementary Material consists of two parts. First, Table S1 lists the 42 *Legionella pneumophila* genomes analyzed. Second, Figure S1 shows the accuracy of one of the programs for picking unique regions surveyed, fur, as a function of input size. MMC
