# Supplementary material for: Fast detection of unique genomic regions
Source: Comput Struct Biotechnol J. 2025 Feb 27;27:843–50. doi: 10.1016/j.csbj.2025.02.025 (PMC11925158; doi:10.1016/j.csbj.2025.02.025)
Supplement: MMC — This Supplementary Material consists of two parts. First, Table S1 lists the 42 Legionella pneumophila genomes analyzed. Second, Figure S1 shows the accuracy of one of the programs for picking unique regions surveyed, fur, as a function of input size. [file mmc1.pdf]

Supplementary Material for *Fast Detection of Unique Genomic Regions*

Beatriz Vieira Mourato & Bernhard Haubold

February 14, 2025

Table S1: The 42 target and neighbor genomes analyzed for target *Legionella pneumophila*; note the six discrepancies between taxonomy and phylogeny in entries 22–27.

#	Accession	Taxonomy	Phylogeny
1	GCA_022870105	target	target
2	GCA_014789345	target	target
3	GCA_003004315	target	target
4	GCA_003004295	target	target
5	GCA_003004275	target	target
6	GCA_003004175	target	target
7	GCA_003004155	target	target
8	GCA_003004135	target	target
9	GCA_003003815	target	target
10	GCA_001766375	target	target
11	GCA_001766355	target	target
12	GCA_001766335	target	target
13	GCA_001766315	target	target
14	GCA_001766295	target	target
15	GCA_001766275	target	target
16	GCA_001592705	target	target
17	GCA_000404245	target	target
18	GCA_000347615	target	target
19	GCA_000306865	target	target
20	GCA_000306845	target	target
21	GCA_000008485	target	target

#	Accession	Taxonomy	Phylogeny
22	GCA_027920565	neighbor	target
23	GCA_000239175	neighbor	target
24	GCA_000092625	neighbor	target
25	GCA_000092545	neighbor	target
26	GCA_000048665	neighbor	target
27	GCA_000048645	neighbor	target
28	GCA_900637585	neighbor	neighbor
29	GCA_900475745	neighbor	neighbor
30	GCA_003004255	neighbor	neighbor
31	GCA_003004215	neighbor	neighbor
32	GCA_003004115	neighbor	neighbor
33	GCA_003003955	neighbor	neighbor
34	GCA_003003865	neighbor	neighbor
35	GCA_003003755	neighbor	neighbor
36	GCA_003003675	neighbor	neighbor
37	GCA_003003595	neighbor	neighbor
38	GCA_001886835	neighbor	neighbor
39	GCA_001886795	neighbor	neighbor
40	GCA_001590695	neighbor	neighbor
41	GCA_001590645	neighbor	neighbor
42	GCA_001590615	neighbor	neighbor

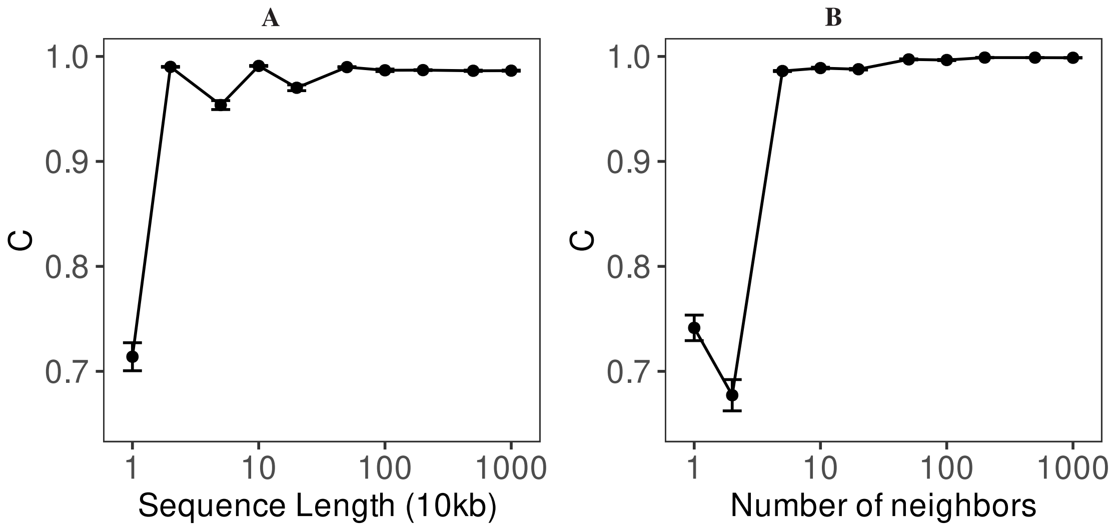

Figure S1: Accuracy of `fur` as a function of sequence length when comparing pairs of sequences (**A**), and neighborhood size (**B**) when comparing 10 kb sequences; shown are mean $\pm$ SEM from 100 simulated samples.
